# Probabilistic Camera Distortion Correction Using Deep Gaussian Processes

**DOI:** 10.3390/jimaging12070296

**Published:** 2026-07-02

**Authors:** Ivan De Boi, Rhys G. Evans, Stuti Pathak, Thomas De Kerf, Marnix Van Soom, Sam Van der Jeught, Helder Araújo, Rudi Penne

**Affiliations:** 1 InViLab, University of Antwerp, Groenenborgerlaan 179, 2020 Antwerp, Belgium; rhys.evans@uantwerpen.be (R.G.E.); stuti.pathak@uantwerpen.be (S.P.); thomas.dekerf@uantwerpen.be (T.D.K.); sam.vanderjeught@uantwerpen.be (S.V.d.J.); rudi.penne@uantwerpen.be (R.P.); 2 AI Lab, Vrije Universiteit Brussel, Pleinlaan 2, 1050 Brussels, Belgium; marnix@ai.vub.ac.be; 3 Institute of Systems and Robotics, Department of Electrical and Computer Engineering (DEEC), University of Coimbra, Rua Sílvio Lima, 3030-290 Coimbra, Portugal; helder@isr.uc.pt

**Keywords:** lens distortion removal, camera calibration, camera distortion correction, deep gaussian processes, fisheye, wide-angle, endoscopy

## Abstract

Accurate lens distortion correction is important for calibration, registration, image stitching, and 3D reconstruction, especially in low-data device-specific settings where disposable or specialised cameras cannot provide large calibration datasets. We address distortion correction for cameras with highly irregular or non-stationary distortion fields, where fixed polynomial models and generic learning-based rectification methods can struggle. We propose a framework based on Deep Gaussian Processes (DGPs) to model the non-linear mapping required for undistortion. The key motivation is that conventional single-layer GPs with stationary kernels must use one global notion of smoothness, whereas DGPs can represent spatially varying behaviour through composed latent mappings while preserving per-pixel predictive uncertainty. This uncertainty can be used to identify or downweight unreliable corrected regions in downstream tasks. We evaluate the method on three real camera datasets with increasing distortion complexity. The full structured acquisitions contain 512 horizontal and 512 vertical line images per camera. These are not thousands of natural calibration images, but they yield up to 29,532, 11,311, and 31,686 detected intersection correspondences for the RPI, Theta, and Pillcam datasets, respectively. This distinction is important for cameras where acquiring many independent images is impractical. The results are assessed using qualitative rectification, uncertainty maps, normalised collinearity errors, and total training time. Polynomial calibration remains strongest for the regular radial RPI distortion, while DGP and DGP2 models show lower normalised collinearity-error distributions than the standard GP and lightweight MLP baselines on the more distorted Theta and Pillcam datasets. For the full datasets, total DGP/DGP2 training times ranged from 2383.50 s to 10092.50 s, reflecting the additional computational cost of probabilistic non-stationary modelling.

## 1. Introduction

**Figure 1:**
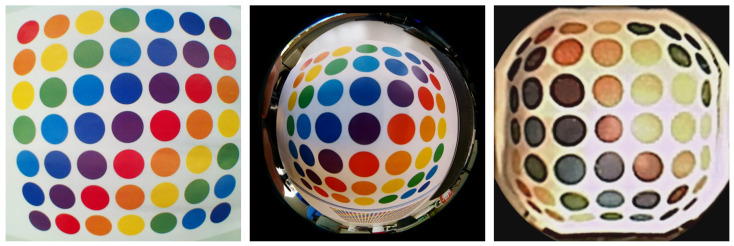
Images of a white piece of paper with coloured circles taken with a wide-angle camera, a 360° camera (half) and a wireless capsule endoscopic camera. Notice how the distortion of the latter appears less consistent with a simple radial polynomial model than the other examples.

The calibration of fisheye lens cameras has seen significant advancements over the last few decades, driven by the increasing prevalence of these cameras in various applications like robotics, automotive systems, and virtual reality [1,2,3,4]. Early calibration methods often relied on simplified models that struggled to accurately represent the severe distortions introduced by wide-angle lenses. Researchers have developed more sophisticated parametric or polynomial models, such as the Kannala–Brandt model [5], which provide more accurate representations of fisheye lens distortion. However, these models still lack the flexibility to handle arbitrary distortions. No matter how many terms are added to a polynomial or how complex the function, a fixed parametric model can only represent a specific type of distortion. Some lenses, such as those used in wireless capsule endoscopy cameras for instance, exhibit highly complex distortions due to manufacturing imperfections, assembly errors (e.g., decentring of lens elements), or even variations in temperature and pressure [2]. These arbitrary or highly irregular distortions do not always fit neatly into a predefined polynomial or radial function. Figure 1 illustrates this concept, showing images of the same scene captured by three different cameras: a wide-angle lens, a 360° camera, and a wireless capsule endoscope.

Commonly used checkerboard calibration methods struggle with images from wide-angle or fisheye cameras, due to the large distortion in the outer regions of the images. This is especially prevalent in endoscopic cameras, which sometimes have low resolutions. Moreover, the process of numbering the found corners in the correct order has also proven challenging, although progress has been made recently by [6].

In recent years, deep learning has emerged as a promising approach for camera calibration [7]. These methods can learn to estimate camera parameters directly from images, potentially reducing the need for specialised calibration patterns. Learning-based methods allow calibration from single images, possibly in uncontrolled environments. A primary drawback of deep learning for fisheye camera calibration is the substantial data requirement. Generating sufficiently large and diverse datasets for training is time-consuming and difficult. Furthermore, these models often struggle to generalise across various fisheye lens types or environments [2].

Taken together, the remaining gap is not simply the absence of another distortion model, but the lack of a method that is simultaneously device-specific, data-efficient, flexible enough for irregular distortion, and probabilistic. Polynomial and parametric models are efficient and interpretable, but their fixed functional form is poorly suited to asymmetric or locally varying distortion. Generic deep learning methods are flexible, but they usually require large representative datasets and may suffer from domain shift when applied to specialised imagery such as endoscopy. Conventional GP-based correction provides uncertainty and can learn from fewer structured observations, but a single stationary covariance function still imposes one global smoothness scale over the full image. This motivates the use of DGPs: they retain the probabilistic uncertainty of GPs while adding compositional flexibility for spatially varying distortion fields.

In this work, we present a device-specific distortion removal method for cases where generic deep learning and polynomial models may be insufficient. Deep learning models trained on generic scenes, such as traffic, houses, or indoor environments, do not necessarily generalise to endoscopic imagery. At the same time, collecting extensive calibration data for an individual endoscopic camera, such as a PillCam™ Crohn’s Capsule camera, is impractical because the device is disposable and has limited battery life. We therefore focus on distortion removal from a limited number of structured calibration images. Wireless capsule endoscopy increasingly relies on automated image analysis, but robustness and data efficiency remain important practical challenges [8,9,10].

The ideal distortion correction method must therefore satisfy three requirements simultaneously. First, it must handle highly irregular, non-stationary distortion fields, where the degree of warping varies non-uniformly across the image plane and cannot be described well by a fixed parametric function. Such non-stationarity can arise from wide-angle lens geometry, decentring or assembly tolerances, manufacturing imperfections, local optical defects, overexposure, and partial visibility of calibration patterns near the image boundary. These effects make the distortion change more rapidly in some image regions than in others, so a single global smoothness assumption becomes restrictive. Second, it must operate with a limited structured calibration acquisition, rather than requiring large and diverse training datasets, which are impractical or impossible to obtain for disposable devices with limited operational lifetime. Third, it should provide per-pixel uncertainty estimates, so that downstream tasks such as image stitching, frame registration, 3D reconstruction, or motion estimation can appropriately downweight regions where the correction is unreliable [11,12,13]. To our knowledge, no widely adopted method jointly addresses irregular non-stationary distortion, low-data device-specific calibration, and per-pixel uncertainty estimation within a single framework.

Once the distortion is corrected or removed in this pre-processing step, the images can be regarded as if they were taken by a pinhole camera. This camera has no effect of lens distortion left in its images. It maps points from the real 3D world to a 2D image plane by tracing rays of light through one central point of projection. This pinhole camera can be easily calibrated by widely known methods [14,15,16]. The need to simultaneously optimise for the distortion parameters and the projection parameters is thus eliminated.

Our proposed method is based on Deep Gaussian Processes (DGPs) [17], which are probabilistic machine learning techniques that extend the capabilities of traditional Gaussian Processes (GPs) by introducing a hierarchical, layered structure. This architectural design draws inspiration from deep neural networks, enabling DGPs to model complex, non-linear relationships within data. We reformulate the distortion removal as a regression problem from an undistorted grid of horizontal and vertical straight lines to the images taken with a wide-angle or fisheye camera. In this context, the DGP offers two main benefits: it can handle non-stationary data (more distortion at the outer regions of the image than in the centre) and it provides an uncertainty estimate (a variance per pixel). The latter can be used in subsequent algorithms such as image stitching or 3D point cloud generation. More uncertain pixels, with a larger variance, can be downweighted accordingly.

A limitation of conventional GP-based distortion correction is that a single GP with a stationary kernel uses one global notion of smoothness across the image plane. This is restrictive for complex lens distortion fields, where the mapping may vary slowly near the image centre but change rapidly and asymmetrically near the boundary. In such cases, the learned kernel hyperparameters represent a compromise between regions with different local lengthscales, which can lead to oversmoothing in highly distorted areas or unnecessary flexibility in smoother regions. DGPs are expected to mitigate this limitation by composing multiple GP layers. The intermediate latent mappings can non-linearly transform the input space before the final regression, allowing the model to represent spatially varying behaviour more naturally than a single-layer GP while retaining probabilistic uncertainty estimates.

To summarise, our main contributions are:

- A Deep Gaussian Process framework for modelling highly irregular, non-stationary camera distortion fields.
- A device-specific calibration approach based on a limited structured acquisition, avoiding the large image collections typically required by generic learning-based rectification methods.
- Per-pixel uncertainty estimates for the corrected image.

The rest of this paper is structured as follows: We provide a comparison to related work in Section 2. We explain the construction of the grid of lines in Section 3, where we also elaborate on Deep Gaussian Processes. In Section 4 we present experiments to assess our method. Finally, we discuss the findings in Section 5 and present our conclusions in Section 6.

## 2. Related Work

Checkerboard calibration generally requires full visibility of the checkerboard pattern, limiting accurate distortion estimation, especially in wide-angle lenses. Their repetitive structure can cause detection failures in challenging lighting or when partially obscured. Accurately ordering corner points in distorted images also presents a significant challenge. Some of these were addressed by [6]. The authors implemented a Gaussian process to learn the mapping from a perfect virtual grid of equidistant corners to the corners detected in the image. This allows for the completion of a partially detected grid. Still, the number of data points is limited to the number of corners. In this work, we will introduce the usage of intersecting lines on a screen to generate more data points (see also Section 3).

Ref. [18] implemented Gray code to overcome the low number of features to perform undistortion. However, in our experiments, we found that this method fails when working with close-range cameras, as the white stripes overwhelm the black ones due to overexposure. In ref. [19] a Gaussian process was used to replace the polynomial describing the distortion itself. This still leads to an iterative process in the camera calibration, resulting in a compromise between distortion and other camera parameters such as focal length, principal point etc. Our method bypasses this iterative process by performing the distortion correction in a pre-processing phase.

A recent implementation by [16] used a Gaussian process to learn the mapping from detected corners in an image to a perfect grid of integer 2D coordinates. This constructs a virtual camera in which all distortion is captured by a Gaussian process. This is suboptimal for distortions that show non-stationarity in the data. For stationary data, which varies evenly everywhere in the input space, the resulting hyperparameter values of the GP should be approximately the same for any subregion of the input space. For non-stationary data, the hyperparameters vary more. This non-stationarity manifests itself in the images by sudden changes in the level of distortion. An example can be seen in Figure 1 on the right. Using an ordinary Gaussian process, as in [16], we obtain results in a set of hyperparameter values that are a compromise across the entire image. We expand on this method by introducing Deep Gaussian Processes to capture this non-stationarity.

The research by [20] proposed a GPU-based method that enables real-time correction of arbitrary geometric distortions learned through deep learning, which lacks uncertainty estimation. Also, their models were trained by learning the relationship from a virtual grid to the real world image pixels. Our method resembles more the inner workings of a camera, which maps from the real world to a virtual image plane.

Recent deep learning-based fisheye and wide-angle calibration approaches learn to correct complex lens distortions end-to-end [7]. Parameter-based networks predict distortion coefficients [21] or dense per-pixel flow fields to warp images back to a perspective view [22]. Self-supervised rectification methods such as SimFIR reduce the reliance on paired distorted and rectified images [23], while generation-based models reconstruct rectified images directly [24]. Diffusion-based models further align synthetic and real fisheye distributions in a shared noise space [25].

Despite their successes, these high-capacity methods typically require vast, diverse training sets and significant computational resources. Their main advantage is that, once trained, they can provide fast single-image rectification and can exploit rich image appearance cues beyond calibration patterns. However, their performance depends on the representativeness of the training distribution, and domain shifts between synthetic or natural-image training data and specialised imagery, such as endoscopic video, can reduce robustness. In contrast, our DGP-based approach is intended as a probabilistic, device-specific correction method that remains practical when only a modest structured calibration set is available. It directly models the geometric mapping from detected line intersections, naturally provides per-pixel uncertainty, and can adapt to irregular non-stationary distortion fields. The disadvantages are that training and dense prediction are slower than for a compact feed-forward network, and that a structured screen-based calibration acquisition is required for each device. Thus, the proposed approach is complementary to generic deep rectification frameworks rather than a direct replacement for them.

## 3. Methods

### 3.1. From Real Image to Virtual Grid

We learn a correspondence from image pixel coordinates (u,v) to virtual grid coordinates (x,y) using a Deep Gaussian Process, as described in Section 3.2. We define a grid of $2^{N}$ rows and $2^{N}$ columns in the virtual (x,y) reference system. We present the camera we wish to calibrate with $2^{N}$ black images in which one column of pixels is made white. The white column is systematically shifted for each subsequent image. We repeat the entire process in an analogous manner for the rows. This results in $2\times 2^{N}$ images. In practice, we recorded a video of an animation showing the white lines in an alternating manner on a computer screen.

**Figure 2:**
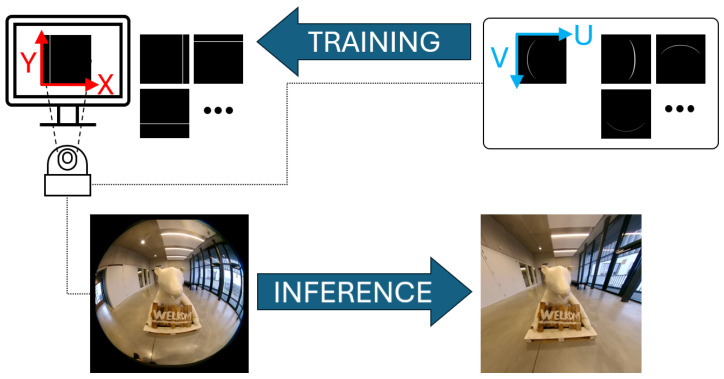
A schematic overview of our proposed method, which learns from uv-coordinates in pixel space to xy-coordinates of intersections of ideal straight lines. At inference, this relationship is used to remove distortion.

For every combination of images of a row and a column, we take the average image and filter out the pixels that are darker than 90 percent of the maximum. This yields a blob of the brightest pixels in the overlapping curved lines. We determine the uv-coordinates of that blob. This gives us a correspondence between the row and column numbers, or xy-coordinates, and the uv-coordinates. Figure 2 provides a schematic overview.

**Figure 3:**
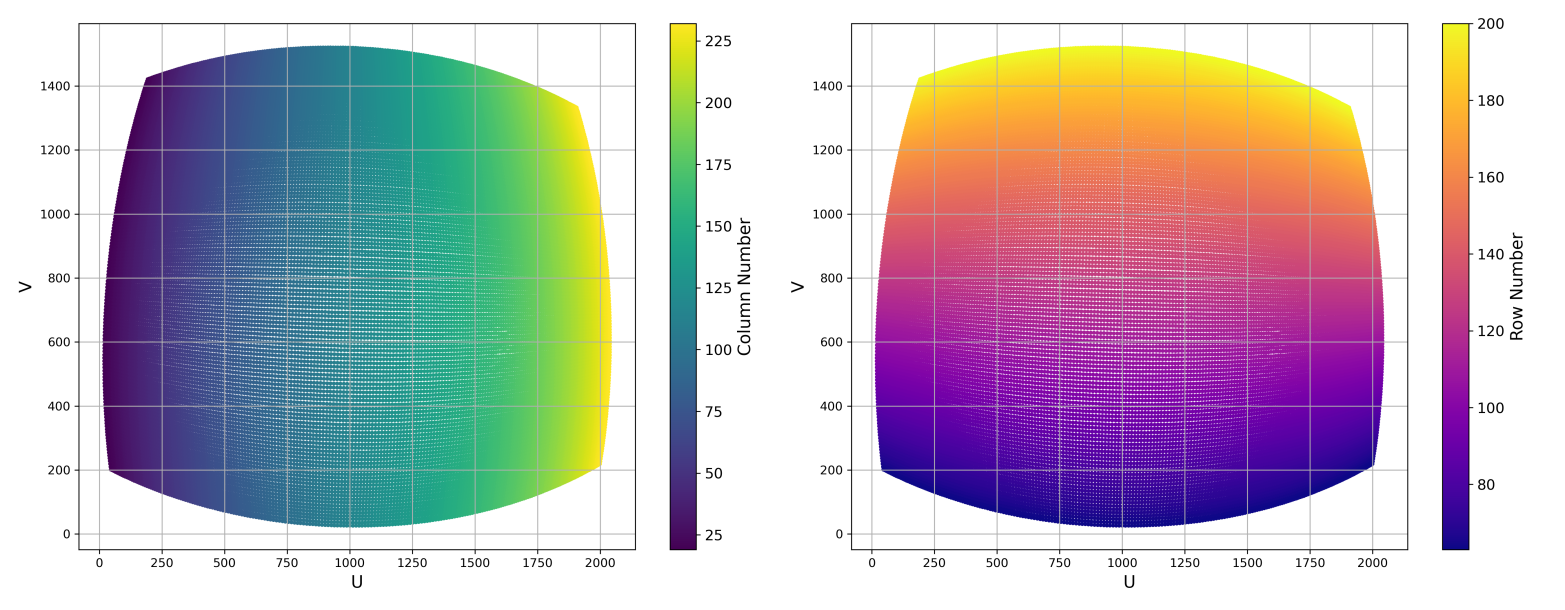
Dataset mapping uv coordinates to rows and columns for the Raspberry Pi camera with wide-angle lens.

**Figure 4:**
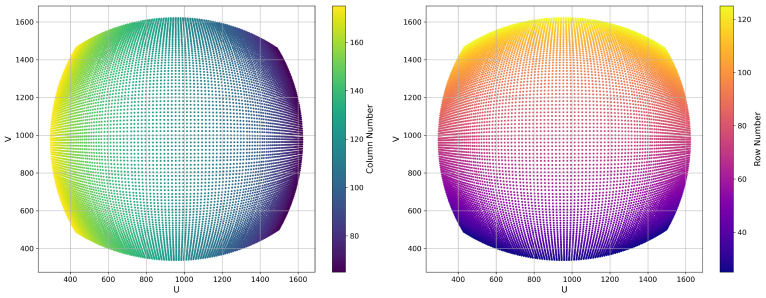
Dataset mapping uv coordinates to rows and columns for the Theta 360° camera, left half.

**Figure 5:**
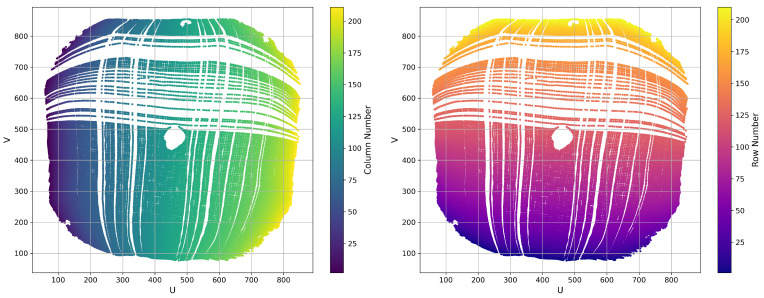
Dataset mapping uv coordinates to rows and columns for the PillCam™ Crohn’s Capsule camera (Medtronic, Minneapolis, MN, USA). The data show non-uniform, asymmetric distortion, missing rows and columns from dropped video frames, and central overexposure from the built-in lights.

**Figure 7:**
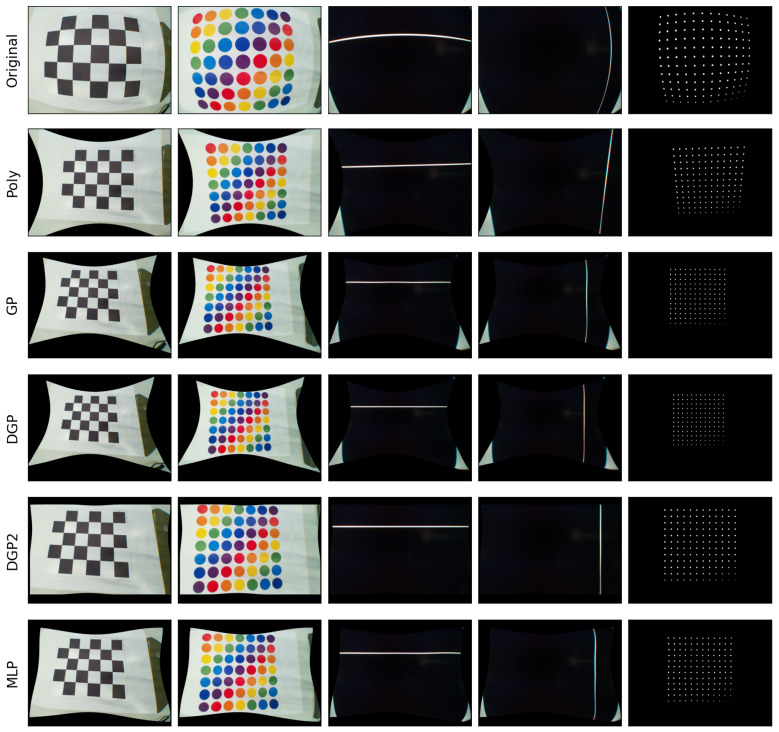
Image comparison for the RPI dataset. The top row shows original distorted images, while subsequent rows display the undistorted results from various models. The columns feature: a checkerboard, coloured circles on a piece of paper, a horizontal and a vertical line, and an overlay of multiple line images of which only the intersections are kept after threshold filtering. This distortion from an ordinary fisheye camera is well represented by the polynomial model, as can be seen in the second row.

**Figure 8:**
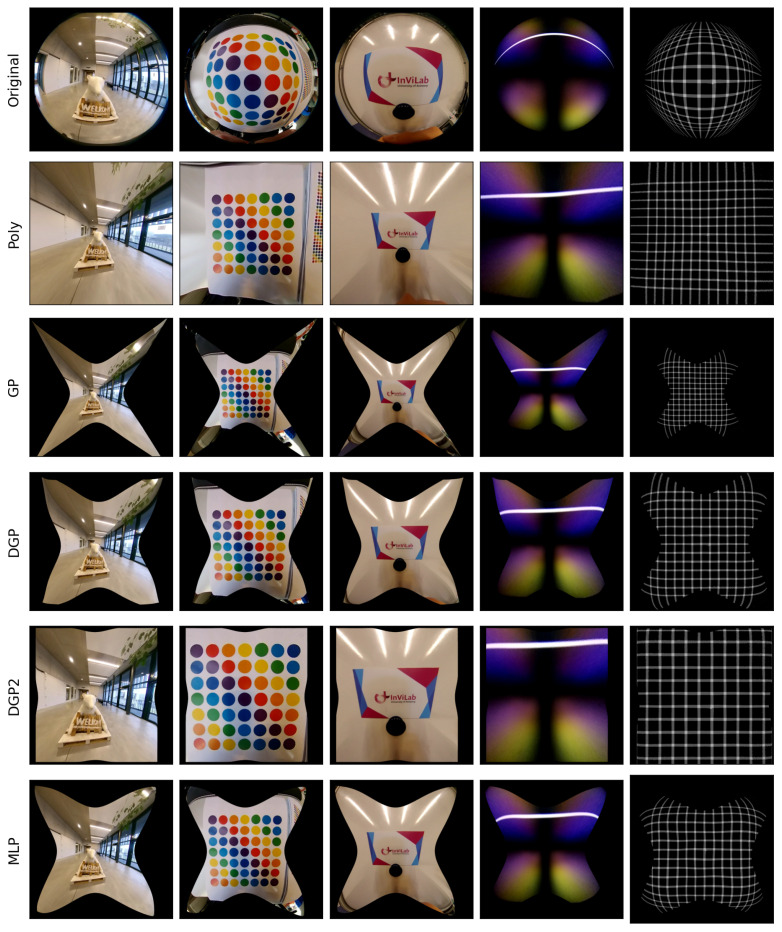
Image comparison for the Theta dataset. The top row shows original distorted images, while subsequent rows display the undistorted results from various models. The columns feature: a statue of a polar bear, coloured circles on a piece of paper, a logo on a business card, a horizontal line, and an overlay of multiple line images. Among the evaluated data-driven models, DGP2 shows favourable qualitative rectification on this dataset. The Poly model shows a slight bend in the horizontal line image. Notice in the last column how the resulting square is more irregular for the Poly model. The GP models show more bending in the outer regions.

**Figure 9:**
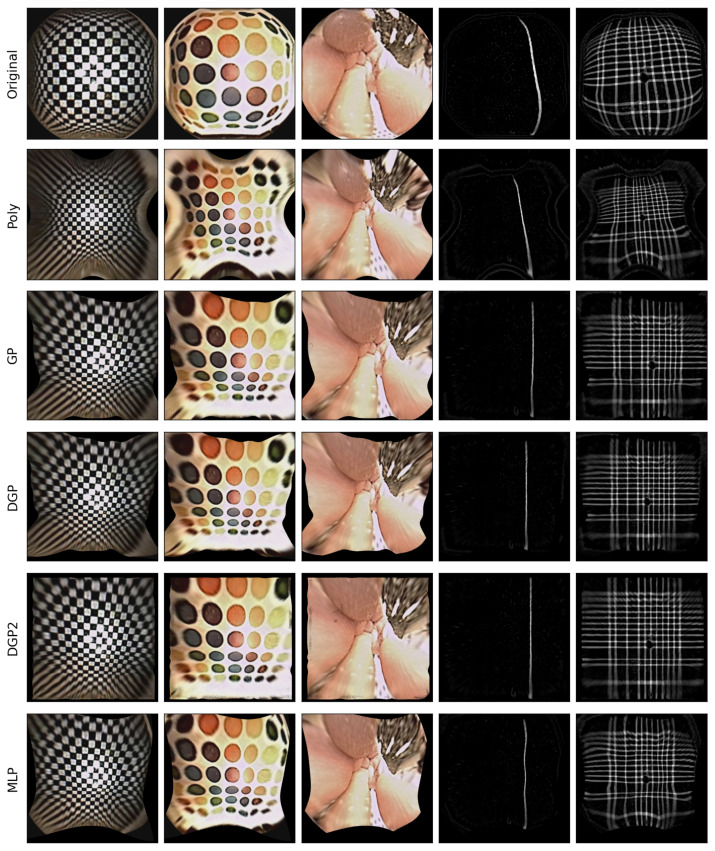
Image comparison for the Pillcam dataset. The figure’s top row shows the original images, while subsequent rows display the undistorted results from various models. The columns feature: a checkerboard, coloured circles on a piece of paper, a view inside a human hand, a vertical line, and an overlay of multiple line images. In this evaluated case, the Poly and MLP baselines do not capture the irregular distortion as well, most notably in the last column.

In this paper, we work with three different datasets. For more details on these, we refer to Section 4.1. Examples of images of the white pixel lines can be found in Figure 7 (third and fourth column), Figure 8 (fourth column), and Figure 9 (fourth column). A visualisation of each of the resulting datasets is given in Figure 3, Figure 4 and Figure 5. Notice how in the centre of the image, the pixels correspond to rows and columns without much change. This change is much more outspoken in the outer regions, where the warping of the lenses is more severe. Moreover, for the PillCam™ Crohn’s Capsule camera, the warping is not regular. There is a distinct transition from the inner region to the outer region.

### 3.2. Deep Gaussian Processes

Gaussian Processes (GPs) provide a robust probabilistic framework for modelling non-linear relationships. An in-depth treatise is given in the book by [26]. In our context, we utilise two GPs to learn a mapping from pixel uv-coordinates to both *x*- and *y*-coordinates of pixels in a virtual perfect grid.

In general, we compose a dataset by taking {X,y} of *n* observations, where $X={\left[\begin{array}{c} x_{1},x_{2},\ldots ,x_{n} \end{array}\right]}^{T}$ is an n×d matrix of *n* input vectors of dimension *d* and $y={\left[\begin{array}{c} y_{1},y_{2},\ldots ,y_{n} \end{array}\right]}^{T}$ is a vector of continuous-valued scalar outputs. These are the training points.

In regression we aim to find a mapping $f:R^{d}\to R$,

(1) $$y=f\left(x\right)+\epsilon ,\;\epsilon ∼N\left(0,\sigma_{\epsilon }^{2}\right),$$

with ϵ being Gaussian noise. In this work, we implement this mapping by a Gaussian process, which is fully defined by its mean m(x) and *covariance function* $k(x,x^{\prime })$. It is generally denoted as $f\left(x\right)∼\mathrm{GP}(m\left(x\right),k\left(x,x^{\prime }\right))$. Typically, the covariance function, or *kernel*, is parametrised by θ, a vector of hyperparameters. By definition, a GP yields a distribution over a collection of functions that have a joint normal distribution,

(2) $$\left[\begin{array}{c} f\\ f_{*} \end{array}\right]∼N\left(\left[\begin{array}{c} m_{X}\\ m_{X_{*}} \end{array}\right],\left[\begin{array}{cc} K_{X,X} & K_{X,X_{*}}\\ K_{X_{*},X} & K_{X_{*},X_{*}} \end{array}\right]\right)$$

where X and $X_{*}$ are the input vectors of the *n* observed training points and the $n_{*}$ unobserved test points, respectively. The vectors of mean values for X and $X_{*}$ are given by $m_{X}$ and $m_{X_{*}}$. The covariance matrices $K_{X,X}$, $K_{X_{*},X_{*}}$, $K_{X_{*},X}$ and $K_{X,X_{*}}$ are constructed by evaluating *k* at their respective pairs of points. In practice, we do not have access to the latent function values directly, which are dependent on the noisy observations y.

Putting it all together, the conditional (predictive posterior) distribution of the GP can be written as:

(3) $$f_{*}|X,X_{*},y,\theta ,\sigma_{\epsilon }^{2}∼N\left(E\left(f_{*}\right),V\left(f_{*}\right)\right)$$

(4) $$E\left(f_{*}\right)=m_{X_{*}}+K_{X_{*},X}{\left[K_{X,X}+\sigma_{\epsilon }^{2}I\right]}^{-1}f$$

(5) $$V\left(f_{*}\right)=K_{X_{*},X_{*}}-K_{X_{*},X}{\left[K_{X,X}+\sigma_{\epsilon }^{2}I\right]}^{-1}K_{X,X_{*}}$$

The kernel is pivotal in shaping the GP model’s smoothness and flexibility. The squared exponential kernel, a popular choice in kernel-based machine learning, is infinitely differentiable, ensuring smooth function outputs. This assumption aligns well with the continuous nature of the lens distortions. This kernel is expressed as:

(6) $$k_{SE}\left(x,x^{\prime }\right)=\sigma_{f}^{2}\exp\left(-\frac{{\left|x-x^{\prime }\right|}^{2}}{2l^{2}}\right),$$

in which $\sigma_{f}^{2}$ is the outputscale factor and *l* the lengthscale that determines the radius of influence of the training points. For the squared exponential kernel the hyperparameters are $\sigma_{f}^{2}$ and *l*. These are learned from the data with empirical Bayes by maximising the marginal likelihood of the data given the hyperparameters.

Alternatively, a different lengthscale parameter for each input dimension can be implemented. This technique is called automatic relevance determination (ARD) and allows for functions that behave differently in each input dimension. For the squared exponential kernel we can generalize Equation (6) as:

(7) $$k_{SEARD}\left(x,x^{\prime }\right)=\sigma_{f}^{2}\exp\left(-\frac{1}{2}\sum_{j=1}^{d}{\left(\frac{\left|x_{j}-x_{j}^{\prime }\right|}{l_{j}}\right)}^{2}\right),$$

in which $l_{j}$ is a separate lengthscale parameter for each of the *d* input dimensions. In this work, there are two input dimensions, namely the *u*- and *v*-coordinates. We implement two independent GPs in parallel: one for (u,v)→x and one for (u,v)→y.

Training a Gaussian Process is learning values for the kernel hyperparameters θ so that they optimise the log *marginal likelihood*, which for Gaussian likelihoods like Equation (1) can be derived in closed form:

(8) $$\log p\left(y|\theta ,X\right)\propto -\frac{1}{2}y^{T}{\left[K_{X,X}+\sigma_{\epsilon }^{2}I\right]}^{-1}y-\frac{1}{2}\log\left|K_{X,X}+\sigma_{\epsilon }^{2}I\right|,$$

where in our case $\theta =\{\sigma_{f},l,\sigma_{\epsilon }\}$. We call Equation (8) the log marginal likelihood, as it is obtained through marginalisation over the latent function *f*. In Bayesian terminology, the marginal likelihood is also called the *evidence* of the model. The first term in Equation (8) is a quadratic data fit term in y and the second term is a complexity penalty. The latter punishes overly complex models that fit the data perfectly but perform badly away from the data points, or models in which the data is explained away through a large measurement noise $\sigma_{\epsilon }^{2}$. In other words, maximizing the (log) marginal likelihood yields a model that explains the data without being overly complex, following the famous Occam’s razor paradigm: the simplest explanation is most likely the best one. A more thorough treatment of Bayesian regularization and model comparison can be found in both the book by [26] and the paper by [27].

Deep Gaussian Processes (DGPs) were first introduced by [17]. They form a hierarchical structure, stacking multiple layers of GPs to capture complex, non-linear relationships within the data. This is comparable to a neural network, but instead of neurons, each layer consists of a single GP. The output of one layer becomes the input for the next, allowing the model to learn increasingly abstract representations of the data. This layered structure enables DGPs to model highly non-stationary data, which means that the covariance between two data points does not depend solely on the distance between them. As can be seen from Figure 1, there is more variation in the corners than in the centre of the data. In other words, the influence of a single data point diminishes faster with distance in the corners than in the centre. This is something an ordinary GP with a squared exponential kernel cannot capture. For a Deep Gaussian Process, we write:

(9) $$f\left(x\right)=f_{n}\left(f_{n-1}\left(\ldots f_{2}\left(f_{1}\left(x\right)\right)\ldots \right)\right),$$

in which each $f_{i}$ is drawn from its own Gaussian process with its own kernel and mean function:

(10) $$f_{i}\left(x\right)∼{\mathrm{GP}}_{i}\left(m_{i}\left(x\right),k_{i}\left(x,x^{\prime }\right)\right).$$

A unifying perspective on how to handle non-stationarity with Gaussian processes is given by [28].

In general, exact inference in Deep Gaussian Processes is intractable. To address this, the model is typically optimized using variational inference. This approach replaces the true posterior distribution over the latent functions (which includes the inducing variables) with a simpler, computationally tractable distribution. The training process involves maximizing the Evidence Lower Bound (ELBO), which is equivalent to minimizing the Kullback–Leibler (KL) divergence between the approximate and true posterior. To address the cubic computational complexity ($O(N^{3})$), inducing points are introduced. They act as a small set of pseudo-observations that summarise the large dataset. More details can be found in [17].

The utility and training stability of DGPs is investigated in [29]. They found that simply increasing the number of layers does not guarantee improved performance. Instead, optimization becomes significantly more challenging due to the highly non-convex nature of the objective function, often leading to poor local optima.

For this reason, we did not assume that a deeper DGP is automatically preferable. Instead, we evaluated a standard GP, a DGP with one hidden layer (DGP), and a DGP with two hidden layers (DGP2). This provides a limited sensitivity analysis on model depth while keeping the optimization problem tractable. The one-hidden-layer DGP is the minimal deep extension of a GP and tests whether a learned latent transformation improves the modelling of non-stationary distortion. The two-hidden-layer DGP tests whether additional compositional flexibility improves the result further. Deeper architectures were not considered, because previous work has shown that increasing the number of DGP layers can make optimization substantially harder without guaranteeing better predictive performance.

The following experiments use this formulation to answer three practical questions: whether the measured distortion fields are non-stationary, whether DGP-based correction improves visual and numerical rectification, and how much calibration data is needed for reliable device-specific correction.

## 4. Experiments

### 4.1. Datasets and Models

We composed three datasets made with different camera systems, each with an increasing level of distortion: (1) A Raspberry Pi with a ZeroCam FishEye lens (Raspberry Pi Ltd., Cambridge, UK) (field of view 170°) and a resolution of 2048 × 1536. (2) A Theta Z1 360° camera (Ricoh Company, Tokyo, Japan) (we only used one of the two 180° camera images) with a 1920 × 1920 resolution. (3) A PillCam™ Crohn’s Capsule camera (field of view 172°) used in endoscopy. We cropped its images from the recorded video to a resolution of 886 × 886 to remove the black borders. The PillCam was chosen to demonstrate the relevance of our findings to a medical device which often shows uncommon distortions. In our experiments, the total number of columns and rows is $2^{9}=512$. These were presented to three different cameras on an Alienware m18 R2 Gaming Laptop (Dell Technologies Inc., Round Rock, TX, USA) with a 18" QHD+ (2560 × 1600) 165 Hz screen and an NVIDIA GeForce RTX 4080 Laptop GPU (NVIDIA, Santa Clara, CA, USA). All datasets are normalised to zero mean and unit variance before training. In what follows, we refer to these datasets as RPI, Theta and Pillcam, respectively. The full training images, checkerboard images, undistorted outputs, trained models, and supporting data are publicly available at https://doi.org/10.5281/zenodo.17721246 (accessed on 28 June 2026).

We compare our proposed Deep Gaussian Process method to the industry standard of fitting a polynomial to capture the distortion as implemented in both MATLAB and OpenCV (Poly). In this work, we used the Camera Calibration Toolbox of MATLAB R2024b implementation. We also evaluate a regular Gaussian process (GP) and a Deep Gaussian Process with one (DGP) and with two hidden layers (DGP2). The (Deep) Gaussian Process models are implemented in *GPyTorch* version 1.15 by [30], a software platform for scalable GPs built on PyTorch.

Finally, we also compare our results to a lightweight multi-layer perceptron (MLP) baseline used as a data-driven neural regressor. This is a shallow, fully connected neural network. It takes 2D coordinates as input and passes them through three contracting hidden layers with sizes 64, 32, and 16, respectively, using the Tanh activation function. The final layer maps the 16-dimensional representation back to a transformed 2D coordinate output.

We do not include a direct experimental comparison with contemporary CNN-, Transformer-, or flow-based rectification frameworks. Such methods are typically trained on large synthetic or natural-image datasets and often assume a generic rectification target, whereas the present work focuses on device-specific geometric correction from a limited structured calibration acquisition. Applying these methods fairly to the present setting would require retraining or fine-tuning them on representative paired distorted/undistorted data for each device, which is precisely the type of extensive data collection that this work aims to avoid. Therefore, our experimental comparison is restricted to polynomial calibration, single-layer GP regression, DGP variants, and a lightweight coordinate-based MLP baseline. The results should be interpreted as evidence for the usefulness of DGPs in the proposed low-data, device-specific setting, not as a claim of state-of-the-art performance against generic deep rectification networks.

Table A1, Table A2 and Table A3 in the appendix provide further details on the evaluated models. Not every presented white line resulted in useful 2D intersection coordinates, because some lines were not fully visible to the camera, were too strongly distorted, or lay partly outside the image. We used $2^{9}=512$ horizontal lines and $2^{9}=512$ vertical lines, yielding $2^{9}\times 2^{9}=262,144$ possible intersections. In practice, more than half of these candidate intersections could typically not be detected reliably, especially in the outer image regions.

The model name in the first column of each table encodes the dataset (RPI, Theta, or Pillcam), the model type (Poly, GP, DGP, DGP2, or MLP), and the number of row and column line images used, following the acquisition procedure described in Section 3. In Section 4.5, we investigate the effect of working with reduced datasets. In this manuscript, the term low-data refers specifically to the number of structured calibration images acquired for a given device, i.e., the number of displayed horizontal and vertical line images. It does not refer to the number of natural training scenes. The actual regression training samples are the detected line-intersection correspondences extracted from these calibration images. Therefore, a relatively limited structured acquisition can still produce many training samples, because each detected horizontal–vertical line intersection provides one correspondence. The term low-data is thus used to describe the acquisition burden on the camera, not the final number of extracted training correspondences. These correspondences are generated from the same controlled sequence of line images and become sparse or unreliable near the image boundary when line detections fail.

As described in Section 3.2, the ordinary GP model suffers from cubic complexity in the number of data points. Therefore, training a GP on the full dataset is not always possible. For the deep GP models, this remains tractable through the use of inducing points. For small datasets, we reduced the number of inducing points to prevent them from exceeding the number of training points. Hyperparameters were selected by maximising the marginal likelihood for the GP models and the evidence lower bound for the DGP models. The squared exponential kernel was used because lens distortion varies smoothly over the image plane. The ARD variant allows different lengthscales in the *u*- and *v*-directions. Learning rates, iteration counts, numbers of data points, and inducing-point counts are reported in Table A1, Table A2 and Table A3. We used full-batch training for the GP and DGP models. Iteration counts were chosen such that both the objective and the training RMSE had converged.

### 4.2. Non-Stationarity

Here, we investigate the non-stationarity of the datasets in a similar manner as done in [28]. Stationary data is characterized by the property that the covariance between two data points is described satisfactorily as depending solely on the distance between them, not on their specific location in the input space. This means that data points that are closer together have higher covariance, and those farther apart have lower covariance. Non-stationary data, however, has a covariance that depends not only on the distance between data points but also on their absolute position in the input space. For regression tasks with non-stationary data, the function being modelled will exhibit greater fluctuations in some regions of the input space than in others.

To assess this property, we train a Gaussian process on subregions of the data and investigate the variance of the resulting values of the hyperparameters. For stationary data, which varies evenly everywhere in the input space, the resulting hyperparameter values should be approximately the same for every training session. For non-stationary data, the hyperparameters vary more.

This provides a quantitative test of non-stationarity: if a single stationary covariance function were sufficient across the whole image, GP hyperparameters estimated from local subregions would remain close to those estimated from the full dataset. Conversely, a large spread in locally estimated outputscale and lengthscale values indicates that different image regions require different covariance behaviour. We therefore use the dispersion of the fitted GP hyperparameters across 100 random subregions as a quantitative indicator of spatially varying, non-stationary distortion.

First, for each of the datasets, we train two GP models on all the data points: one for the mapping from uv to *x* and one for the mapping from uv to *y*. We implement a non-ARD squared exponential kernel, meaning all $l_{j}$ in Equation (7) are equal. This yields a single value for the outputscale $\sigma_{f}^{2}$ and the lengthscale *l* for each of the two GPs. These values serve as the means for our initial values for the hyperparameters in subsequent training sessions. The actual initial values are sampled uniformly between plus and minus 10% of those means. Next, we perform 100 training sessions on both all the data points and on random subregions of the dataset. These consist of input space patches that are 25% of the total input space in both the *u*- and *v*-directions.

**Figure 6:**
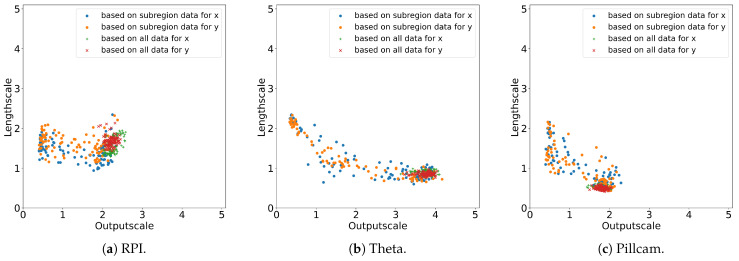
Outputscale and lengthscale values obtained from repeated standard GP fits on the full dataset and on randomly sampled local subregions. Each panel shows the fitted hyperparameters for the mappings to *x* and *y* coordinates for one camera dataset. Hyperparameters estimated from the full dataset cluster tightly, whereas subregion-based fits are more dispersed, especially for the Theta and Pillcam datasets, indicating spatially varying distortion and non-stationary behaviour.

We depict the 100 resulting outputscales and lengthscales for the GPs for *x* and *y* and for both the full dataset and the ones only trained on subregions in Figure 6. Notice how the results are clustered when trained on all the data and more spread out when trained on subregions of the data, indicating non-stationarity.

The separation between the compact full-dataset clusters and the broader subregion-based distributions is most pronounced for the Theta and Pillcam datasets. This quantitatively supports the assumption that the distortion field is not described equally well by a single global lengthscale everywhere in the image. It also motivates the use of DGPs, which can represent spatially varying behaviour through latent transformations, while the standard GP must fit one global covariance structure.

### 4.3. Qualitative Results

In this work, we do not have access to images that represent a ground truth, as we do not train any model on synthetic data. We are interested in a lens distortion removal method for a specific camera, the actual device, and not a general solution suitable for a range of cameras. Additionally, we do not want to rely on a large set of images, possibly thousands, taken with that camera. For disposable cameras, such as the Pillcam with limited battery life, this would reduce its useful remaining lifetime significantly. Moreover, when working with deep learning solutions, the resulting method depends on the nature of the training set images, e.g., urban scenes, indoor or wildlife images. These are not representative for other environments such as endoscopy. Finally, standard mean or RMS reprojection errors cannot be evaluated in the conventional sense because our method does not estimate a parametric camera model with intrinsic and extrinsic parameters. Moreover, reliable checkerboard corner detection (the prerequisite for reprojection-error computation) is precisely one of the limitations our approach is designed to overcome in highly distorted images.

The absence of an external ground-truth undistorted image is therefore a limitation of the real-camera experimental setting. Instead of reporting absolute pixel-wise ground-truth error, we evaluate geometric consistency using the straight-line structure that should be recovered after undistortion. The normalised collinearity error used in Section 4.5 serves as a practical proxy for distortion-correction error: after successful correction, the projected horizontal and vertical line patterns should form a regular grid with intersections close to those of fitted straight lines. This metric does not replace synthetic ground truth, but it allows the evaluated methods to be compared on real camera data without assuming a particular parametric distortion model.

We assess the fitness of our camera distortion correction using two methods: a qualitative comparison and a numerical analysis. This section presents a purely qualitative comparison of the undistorted images generated by the Poly, GP, DGP, DGP2, and MLP methods. The next section details the numerical assessment in the form of a collinearity test. This metric is useful when the line structure remains recoverable, but it becomes less informative in severe failure cases where the fitted row and column lines are themselves unreliable.

Figure 7, Figure 8 and Figure 9 display a selection of undistorted images. We only included images undistorted by methods trained on the full dataset. In each of these images, we observe a slight bend in the Poly undistorted image (second row) of the straight line (fourth column). The data-driven methods appear to handle the distortion better in the centre, but worse in the outer regions. The reason for this is that both the GP models and the MLP models are data-driven. In those outer regions, the data is more noisy, as is it harder for our proposed method to detect the lines and the intersection of those lines. The distortion is so severe that the lines are very narrow or even not visible. The Poly model, which relies on an underlying geometrical description of the distortion, handles the outer regions better, as it can extrapolate by the polynomial for which the coefficients have been learned. The downside for this Poly method is when the polynomial does not capture the distortion properly, as can be observed for the Pillcam dataset in Figure 9.

### 4.4. Pixel Uncertainty

A major benefit of our proposed method is the uncertainty estimate that is inherent to working with Gaussian processes. Not only do we get xy-prediction for every original uv-pixel coordinate, we also obtain a posterior variance for that prediction. In other words, we have information about how certain we are of that prediction.

**Figure 10:**
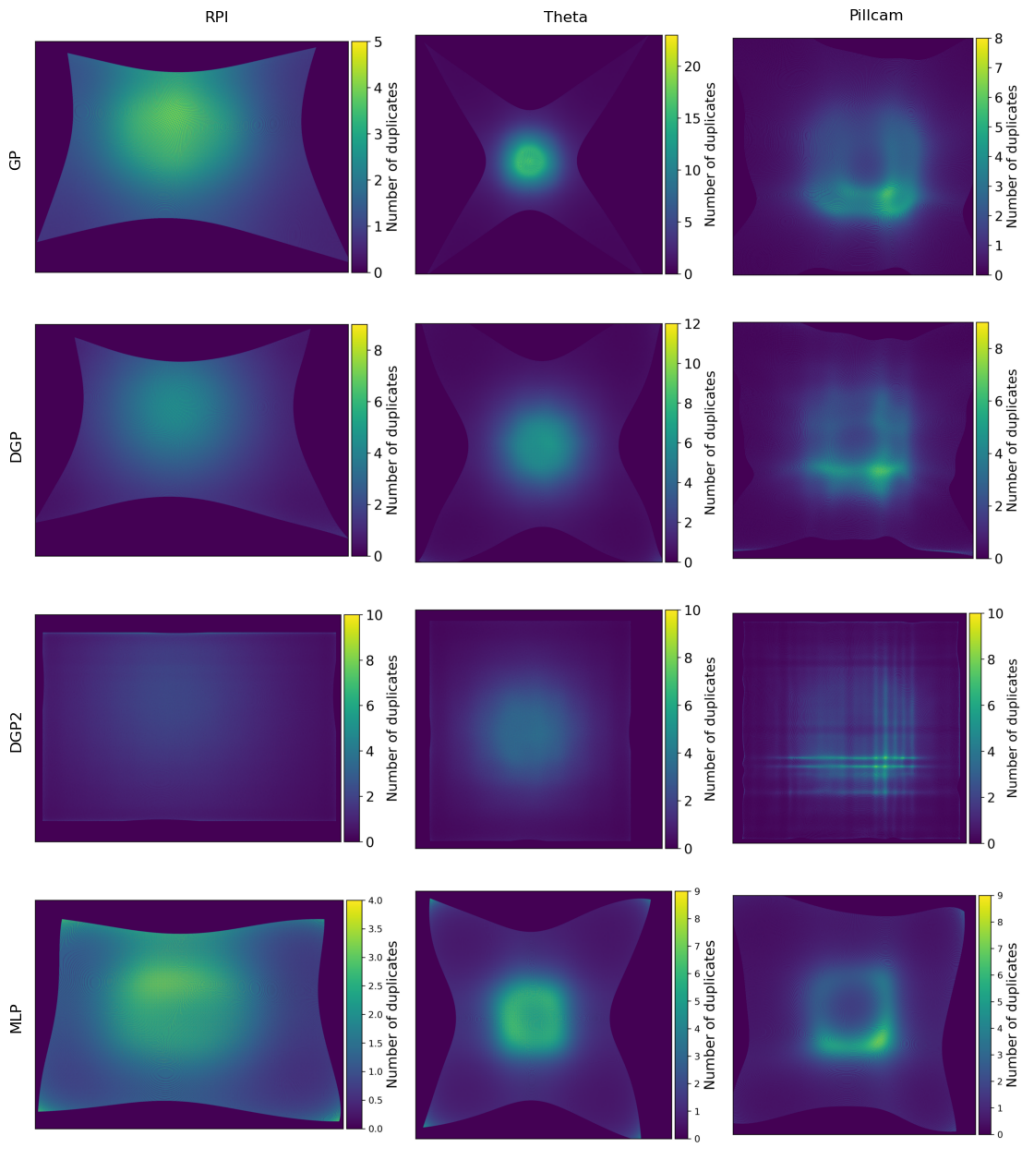
Number of source pixels mapped to each destination pixel after distortion correction for the full datasets. Columns correspond to the three camera datasets and rows to the evaluated data-driven models. Brighter regions indicate destination pixels that receive multiple source pixels and therefore require duplicate-resolution by averaging the contributing pixel values. Dark regions indicate pixels that receive few or no mappings, typically near boundaries or in areas where the learned transformation extrapolates beyond the observed line intersections. The spatial pattern of duplicates reflects how each model redistributes pixels during undistortion: regular radial datasets show smoother central accumulation, whereas the Pillcam dataset produces more irregular duplicate patterns because of its asymmetric distortion and missing line detections.

After training the models, we use their predictions to map every integer uv-coordinate to a new integer xy-coordinate and construct the undistorted image in xy-space. Two practical issues arise during this forward mapping. First, some destination pixels receive no source value and therefore remain empty. We fill these holes using a weighted average of neighbouring pixel values, with weights calculated as the inverse of the neighbours’ uncertainties, i.e., their posterior variances. This emphasizes information from the most certain predictions. Second, especially near the centre of the undistorted image, several source uv-coordinates may map to the same destination xy-coordinate. For these duplicate assignments, the destination pixel value is computed as a weighted average of the contributing source values. Again, the weights are based on the inverse of the accompanying uncertainties, ensuring that the most confident predictions contribute most strongly to the final pixel value. The MLP model, which lacks posterior uncertainty, uses a uniform weight of 1 for all predictions when resolving both holes and duplicate assignments, making this post-processing step less informative. For the models trained on the full datasets, we provide a depiction of the duplicate assignments in Figure 10. Notice how the tails or corners contain more missing pixel values, whereas the centres contain more duplicate assignments.

**Figure 11:**
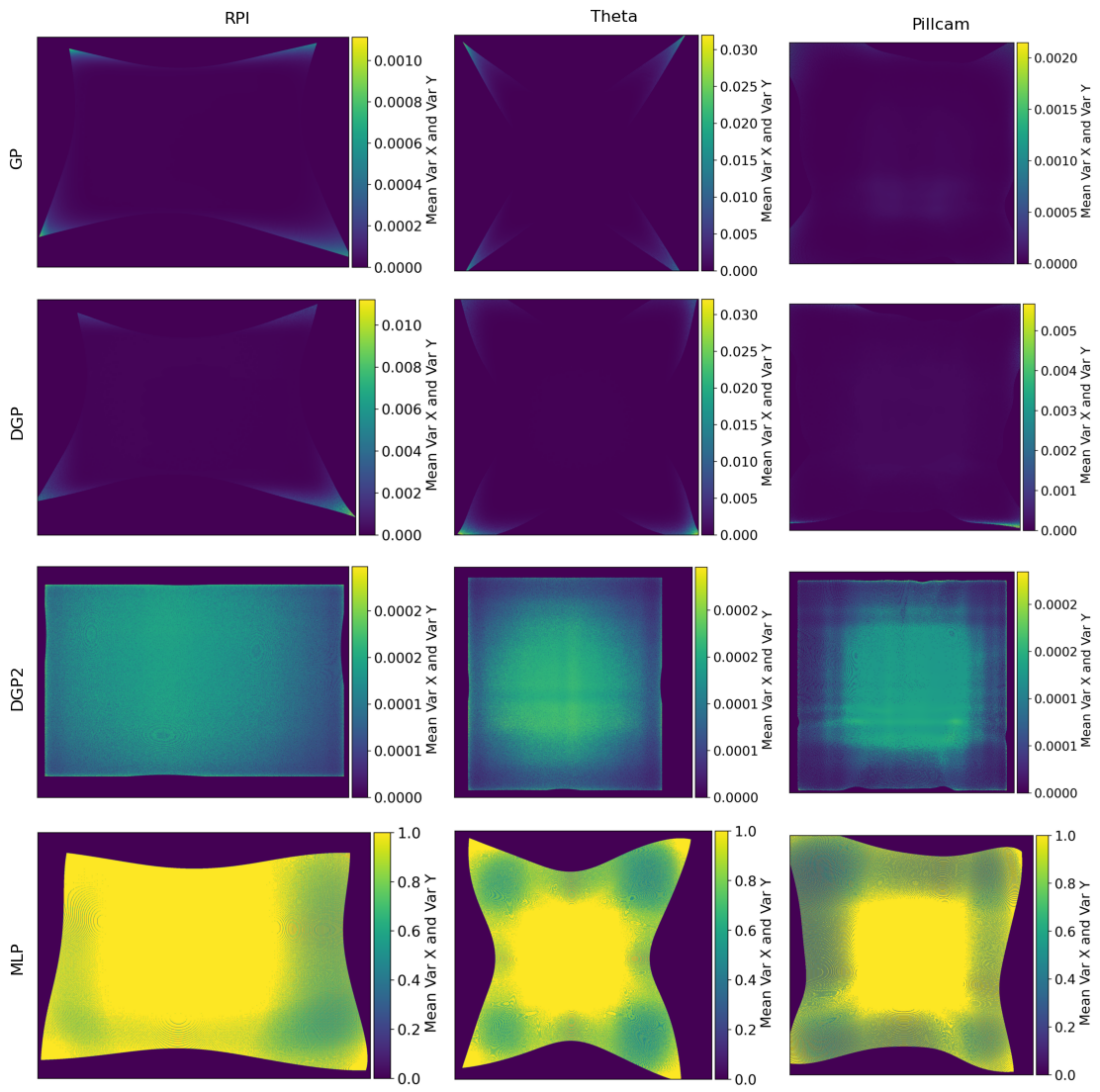
Posterior uncertainty per destination pixel for the full datasets. Brighter regions indicate larger predictive variance and therefore lower confidence in the corrected pixel location. The GP and DGP models provide predictive variances that increase mainly near image boundaries, where line detections are sparse or unreliable. The MLP baseline is deterministic and does not produce posterior uncertainty. Its displayed values are therefore either a constant weight of 1 for pixels that receive a mapping, or empty/no value for pixels to which no source pixel is mapped.

Furthermore, uncertainty acts as a built-in quality control mechanism. We leverage the uncertainty estimate to threshold and cut off regions where the predicted pixel locations are potentially incorrect or possess low confidence. This is visible in the visual comparisons (Figure 7, Figure 8 and Figure 9), where the GP models, while extrapolating poorly away from the data, simultaneously reveal this low confidence via increasing uncertainty. Conversely, the Polynomial (Poly) model can extrapolate reasonably well if the distortion perfectly fits the polynomial function (RPI and Theta datasets) but fails and provides no certainty measure when it does not (Pillcam dataset). The MLP model suffers from both poor extrapolation and the lack of an uncertainty measure. For the models trained on the full datasets, we provide a depiction on the uncertainties in Figure 11. The resulting uncertainty shown is the mean of the uncertainty for the *x*- and the *y*-prediction.

**Figure 12:**
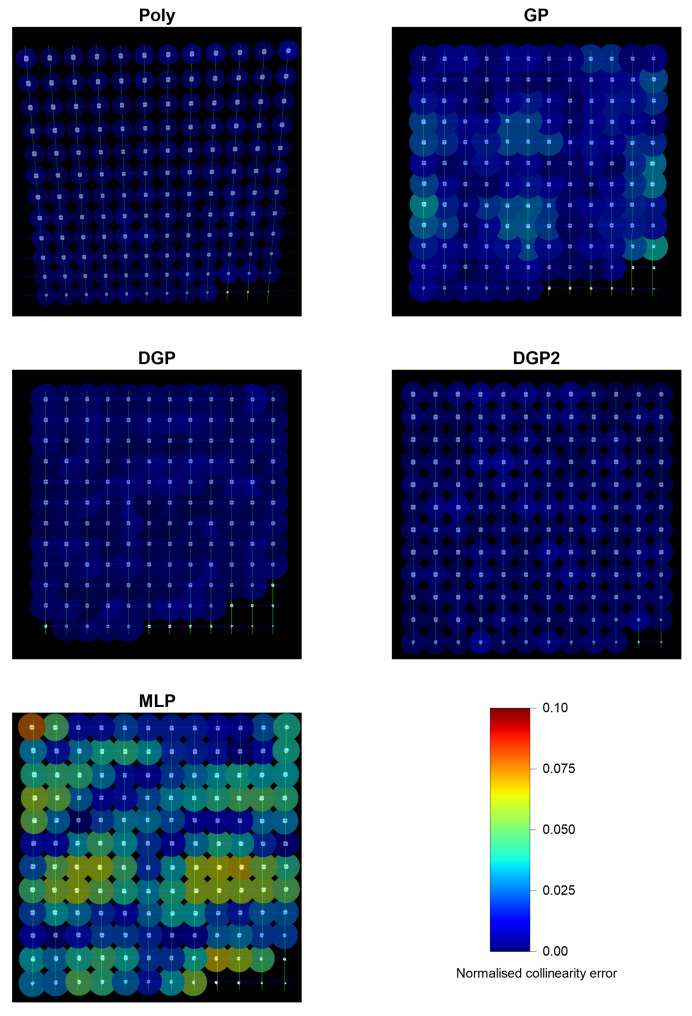
Normalised collinearity errors for the undistorted RPI line grids. The polynomial model shows favourable error distributions for this regular radial-distortion dataset.

**Figure 13:**
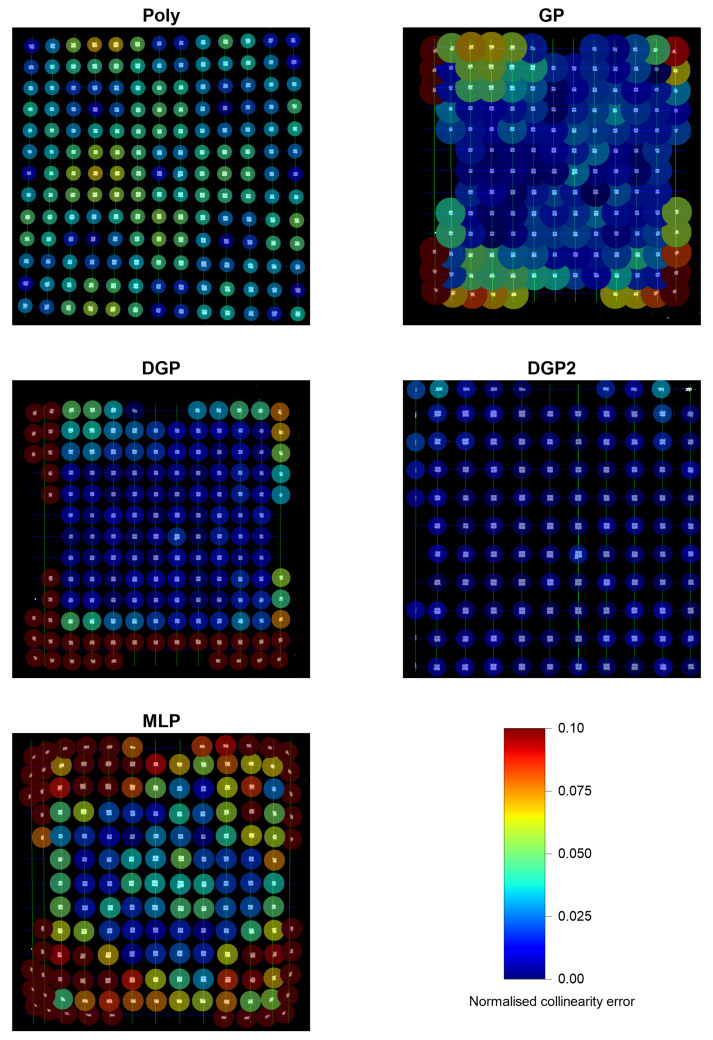
Normalised collinearity errors for the undistorted Theta line grids. DGP and DGP2 reduce the peripheral error patterns relative to the standard GP and MLP baselines.

**Figure 14:**
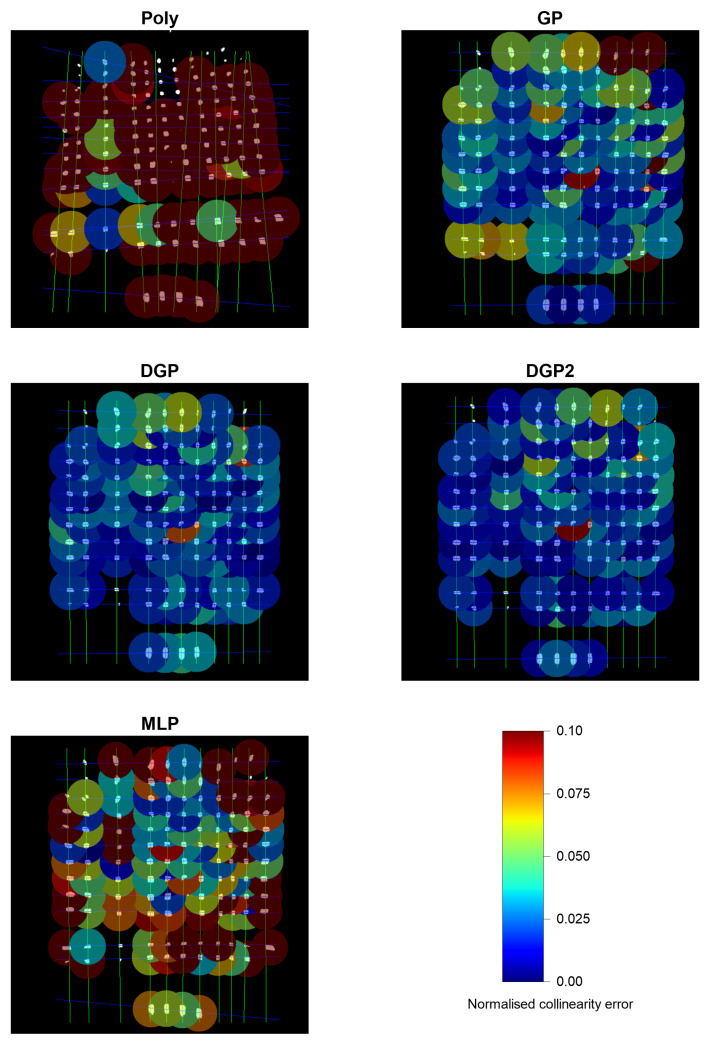
Normalised collinearity errors for the undistorted Pillcam line grids. The DGP-based models provide the most consistent correction among the evaluated models for this irregular distortion field.

To quantitatively assess whether predictive uncertainty reflects correction reliability, we compared the posterior variance with the normalised collinearity error used in Section 4.5. For each GP, DGP, and DGP2 model trained on the full datasets, the predictive variance can be sampled at the corrected line-grid intersection locations and paired with the corresponding normalised distance to the fitted ideal grid. This provides a direct uncertainty-error comparison at the same geometric locations. In the evaluated images, the largest predictive variances occur mainly near the peripheral regions where line detections are sparse or unreliable, which are also the regions where the largest collinearity errors are observed in Figure 12, Figure 13 and Figure 14. This quantitative pairing supports the interpretation of the uncertainty maps as indicators of correction reliability. We do not interpret the posterior variance as an absolute calibrated pixel-error value, because the real camera experiments do not provide an independent ground-truth undistorted image. Instead, the collinearity error provides a practical geometric error proxy for assessing whether high uncertainty coincides with unreliable correction.

### 4.5. Numerical Results

In this section, we provide a numerical assessment of our camera distortion correction method. Overall, we measure how well the undistorted images of straight lines show lines that are actually straight. To this end, we use the image in Figure 7, Figure 8 and Figure 9 in the top row on the right. This is the result of an overlay of several images of straight lines. We distil the coordinates of the intersections of those lines and fit a straight line for every row and column in the lattice of intersections. We calculate for every measured intersection the distance to the intersection of the fitted row and column lines. For a perfect grid, these should coincide. We also normalise these distances by dividing by the median of the distances between the row lines and the column lines. This removes the dependency on the resolution. We thus obtain for every measured intersection a unitless number which indicates the deviation from a perfect lattice.

For every dataset and every model, we depict the errors (the distances to an intersection of an ideal grid) by means of coloured filled in circles in Figure 12, Figure 13 and Figure 14. The colour scale is between blue for small errors to red for errors of 0.1. This measure is unitless and the same for all images. The colour scale is capped at a normalised error of 0.1 to preserve contrast among the relevant low-error regions. Values above this threshold are displayed with the highest colour value.

This collinearity metric specifically evaluates whether straight calibration lines remain straight and mutually consistent after correction. It does not fully measure local scale preservation, aspect-ratio preservation, angular accuracy, or area distortion. In other words, a method could make lines appear straight while still introducing local magnification or compression errors. Moreover, the metric is not a standard camera-calibration metric such as mean reprojection error, RMS reprojection error, calibration residual, or geometric distortion error. Because no independent metric ground-truth image or calibration object with precisely known 3D geometry is available for the real cameras used here, we interpret the collinearity error as a geometric proxy for distortion-correction quality rather than as a complete validation of all metric properties of the corrected image.

**Figure 15:**
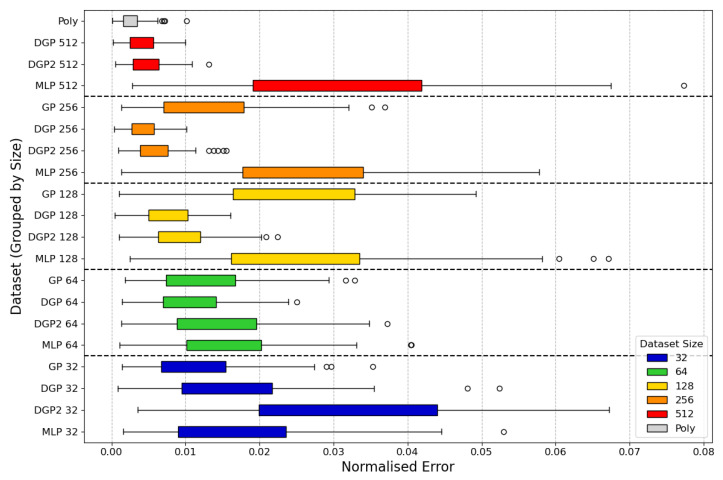
Overview of the normalised distances to the corresponding intersection of an ideal grid for the RPI dataset. Notice how the Poly model (top row) shows the lowest normalised distances among the evaluated models.

**Figure 16:**
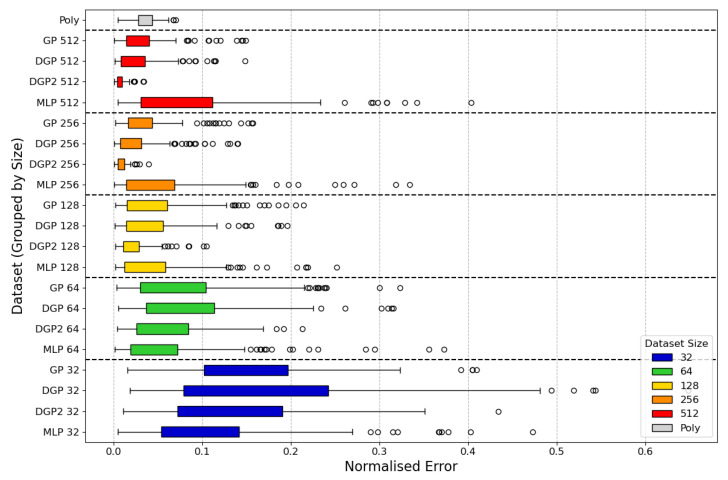
Overview of the normalised distances to the corresponding intersection of an ideal grid for the Theta dataset. The DGP2 model shows lower normalised distances than the other evaluated models when sufficient data are available.

**Figure 17:**
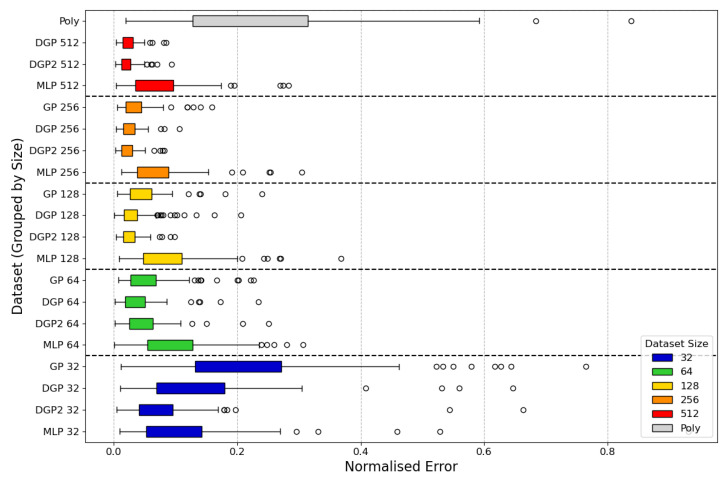
Overview of the normalised distances to the corresponding intersection of an ideal grid for the Pillcam dataset. Both the DGP and DGP2 models show lower normalised distances than the GP and Poly models in this evaluation.

We also investigate how the number of data points in the dataset affects the overall results, which addresses the crucial question of how many images are necessary for device calibration. The results shown in Figure 7, Figure 8 and Figure 9 utilize the full datasets. We additionally create reduced datasets by selectively keeping every second, fourth, eighth, and sixteenth row and column, effectively removing data with each step. Table A1, Table A2 and Table A3 provide an overview of all models respectively. We provide boxplots for the resulting normalised distances of the models RPI, Theta and Pillcam trained on all these variants in Figure 15, Figure 16 and Figure 17 respectively.

The error distributions visualized in these boxplots are consistent with the qualitative assessments of the resulting undistorted images. Overall, DGP and DGP2 achieve lower errors than the standard GP and the lightweight MLP baseline on the more distorted Theta and Pillcam datasets. For the RPI dataset, the Poly method performs best because its underlying geometric model is well-suited to capture the camera’s regular radial distortion. For the challenging Pillcam dataset, where the distortion is irregular and appears less well described by a radial polynomial, DGP and DGP2 provide the most consistent correction among the evaluated models on this dataset. Finally, the presence of black circles, which signify outliers, within the boxplots correlates directly with the poorly corrected or highly uncertain regions, often depicted as red filled-in circles in the outer regions of the data-driven models’ visualizations in Figure 12, Figure 13 and Figure 14.

We also see that the performance of all data-driven methods degrades strongly when not enough data is presented, e.g., when working with only 64 or even 32 images of horizontal and vertical lines in the dataset. Conversely, increasing the dataset from 256 to 512 images results in marginally better performance at the cost of significantly more training time. See also Table A1, Table A2 and Table A3 for details on the time per iteration as a function of the size of the dataset.

While convenient, the proposed numerical metric fails for images that are not properly undistorted because the row and column lines cannot be accurately determined, leading to major outliers. While we include these numerical results for completeness, they should not be interpreted in a relative manner compared to the data from properly undistorted images. An example of such failure can be seen in Figure 14, which shows the Poly method applied on the Pillcam dataset. It is clear from the white pixels that this is not a properly undistorted image.

### 4.6. Training Time

The computational complexity of the proposed models is an important consideration. Table A1, Table A2 and Table A3 report the number of iterations and the time per iteration for all reduced and full datasets. Because the number of iterations differs between models, Table 1 additionally reports the resulting total training time for the full-dataset experiments, computed as the number of iterations multiplied by the measured time per iteration.

The MLP models exhibit the fastest total training time across all datasets (RPI, Theta, and Pillcam). This speed comes at the cost of lacking the uncertainty estimation inherent to probabilistic models like Gaussian Processes. The ordinary Gaussian Process (GP) models, while providing uncertainty, suffer from cubic computational cost in the amount of data points. This makes training on the full RPI and Pillcam datasets computationally intractable or highly time-consuming for the standard GP implementation, which is why those full GP entries are omitted. The proposed Deep Gaussian Processes (DGP and DGP2) address this complexity challenge by employing inducing points. These pseudo-observations summarise the large dataset, making the training process tractable even for the full datasets. While the DGPs’ total training time is substantially higher than that of the MLP models, their overall training remains feasible as an offline device-specific calibration step, and they offer the significant benefit of providing per-pixel uncertainty estimates and better performance on non-stationary data compared to the standard GP and MLP. The main contributor to the overall training time is the number of data points, not only the number of hidden layers in the DGP architecture. For example, full-dataset DGP and DGP2 training times are of the same order for RPI and Theta, while the Pillcam DGP2 model is slower because it uses more inducing points.

In summary, the experiments show that polynomial correction remains strong when the distortion follows a regular radial pattern, as in the RPI dataset. For stronger or more irregular distortions, especially the Pillcam dataset, the DGP models provide better correction than the GP, MLP, and polynomial baselines while preserving useful per-pixel uncertainty estimates. The evaluated results suggest that moderate calibration sets can be sufficient in these settings, whereas very sparse line sets degrade all data-driven methods.

## 5. Discussion

The proposed method is particularly well suited to disposable medical devices because it achieves useful correction with a moderate amount of calibration data in the evaluated settings. The experiments show that performance drops sharply when the dataset is too small, such as fewer than 64 images, while larger datasets provide diminishing returns relative to their additional training cost. By performing distortion correction as a pre-processing step, the framework also simplifies the overall calibration process by eliminating the need to simultaneously optimize for both distortion and projection parameters. Overall, this research establishes DGPs as a powerful tool for achieving precise, automated image rectification in specialized imaging applications.

The experiments were designed to address the three main claims of the paper. Non-stationarity is evaluated through the dispersion of GP hyperparameters fitted on random image subregions, as described in Section 4.2. The low-data setting is evaluated by training on progressively reduced sets of structured line images and comparing the resulting collinearity errors. Uncertainty quantification is evaluated by visualising posterior variance and by relating high-uncertainty regions to high collinearity-error regions. Nevertheless, these experiments do not constitute a full metric calibration benchmark with independent ground truth. In particular, the current line-based metric validates straightness and grid consistency, but does not completely quantify local scale distortion, aspect-ratio preservation, angular relationships, or downstream calibration and reconstruction accuracy. A future extension with synthetic data, an independently measured calibration target, or a calibration object with known geometry would allow standard metrics such as mean reprojection error, RMS reprojection error, calibration residuals, geometric distortion error, and 3D reconstruction accuracy to be evaluated more directly after distortion correction.

The limited improvement from DGP to DGP2 is also consistent with the known behaviour of Deep Gaussian Processes. As discussed by [29], adding layers to a DGP does not necessarily improve performance, because the variational optimization problem becomes more difficult and may converge to poorer local optima. In our experiments, one hidden layer already provides a latent transformation that can capture the main spatial variation in the distortion field. The second hidden layer adds compositional flexibility, but this additional capacity only yields small improvements in the evaluated collinearity errors while increasing computational cost, especially for the Pillcam dataset where more inducing points were used. This suggests that, for the distortion fields considered here, DGP2 may be close to a diminishing-returns regime: it can improve some cases, but the gain is not proportional to the added complexity.

However, we do acknowledge some limitations. The overall accuracy is highly dependent on the initial detection of straight horizontal and vertical lines within the distorted image. This detection is most difficult in the severely distorted, narrow outer regions, where the training correspondences are sparse and less reliable. The data-driven Gaussian-process models also do not extrapolate well beyond regions where lines were detected. This is especially important near image boundaries: when calibration lines become too thin, saturated, partially outside the field of view, or invisible, the learned mapping is forced to infer the correction outside the observed support. In these regions, the resulting rectification can contain missing pixels, duplicate mappings, or unstable geometric corrections. The posterior uncertainty helps identify such unreliable regions, but it does not by itself recover accurate geometry where no reliable training correspondences exist. Finally, the method requires a screen-based calibration acquisition and was evaluated on three camera systems. Broader validation is still needed for other non-radial distortions, camera resolutions, and acquisition conditions. A further limitation is that we did not benchmark against a full modern CNN-, Transformer-, or flow-based rectification framework. Such a comparison would be valuable for assessing competitiveness against generic state-of-the-art rectification methods, but it would require a carefully matched training protocol and representative paired data for the device-specific low-data setting considered here.

In future work, we aim to investigate the application of more intricate kernel functions, such as the Matérn kernel [17], to better characterise potential discontinuities in lens distortions, e.g., a fault in the glass of the lens or even a crack.

Another key area of investigation is the integration of the per-pixel uncertainty into 3D Gaussian Splatting, image stitching or 3D point cloud generation. By leveraging this uncertainty, we can refine the optimization process. Specifically, pixels with lower uncertainty can be weighted accordingly. Conversely, pixels with higher uncertainty, indicative of potential inaccuracies, can be de-emphasised, thus minimising rendering artefacts.

This work focused on real world cameras with various types of distortion: a camera with a wide-angle lens with heavy radial distortion, a 360 camera with very severe radial distortion and a wireless endoscopic capsule camera with irregular radial distortion. We plan to expand the scope of distortion models beyond the radial types examined here. A fruitful avenue is the utilization of synthetic data to systematically explore complex and non-standard distortions, such as mustache or thin prism aberrations. The mathematical control afforded by synthetic data will enable a precise analysis of model performance under non-stationary distortion fields. Furthermore, this approach will facilitate the rigorous assessment of the model’s robustness against highly localized deformations (e.g., those induced by manufacturing faults or defects within the lens glass).

## 6. Conclusions

We proposed a Deep Gaussian Process framework for device-specific camera distortion correction in low-data settings. The method is especially useful for irregular distortions, such as those observed with the PillCam™ Crohn’s Capsule camera, where polynomial models are less reliable. By correcting distortion as a pre-processing step, the approach simplifies subsequent pinhole-camera calibration and provides per-pixel uncertainty estimates for downstream tasks.

Overall, the results on the evaluated datasets suggest that DGP-based correction is most beneficial in device-specific low-data settings with irregular or non-stationary distortion, whereas classical polynomial models remain highly effective when the distortion is well described by a regular radial form. Adding a second hidden layer gave only limited additional benefit relative to its training cost.

## Figures and Tables

**Table 1:** Total training time for the full-dataset models. Values are computed from the iteration counts and per-iteration times in Table A1, Table A2 and Table A3.

Dataset	GP 512	DGP 512	DGP2 512	MLP 512
RPI	–	7123.75 s	4046.50 s	0.30 s
Theta	339.05 s	2383.50 s	2480.00 s	2.80 s
Pillcam	–	7023.96 s	10,092.50 s	0.35 s

## Data Availability

The data presented in this study are openly available in at https://doi.org/10.5281/zenodo.17721246, accessed on 28 June 2026. The code is available upon reasonable request from the corresponding author.

## Appendix A. Details of the Data-Driven Models per Dataset

**Table A1:** Overview of the data-driven models for the RPI dataset, their properties and time to train.

Dataset Model $2^{N}$	Row and Column Nr Divisible by	Nr of Datapoints	Nr Iterations	Learning Rate	Time Per Iteration (s)	Nr Inducing Points
RPI GP 32	16	117	500	0.010	0.0188	NA
RPI GP 64	8	486	500	0.010	0.0170	NA
RPI GP 128	4	1890	500	0.010	0.6950	NA
RPI GP 256	2	7383	250	0.010	9.9974	NA
RPI DGP 32	16	117	2500	0.020	0.0485	50
RPI DGP 64	8	486	2500	0.020	0.0515	50
RPI DGP 128	4	1890	2500	0.020	0.0663	50
RPI DGP 256	2	7383	2500	0.020	0.2570	50
RPI DGP 512	1	29,532	2500	0.020	2.8495	50
RPI DGP2 32	16	117	2500	0.010	0.0453	50
RPI DGP2 64	8	486	2500	0.010	0.0999	50
RPI DGP2 128	4	1890	2500	0.010	0.1189	50
RPI DGP2 256	2	7383	2500	0.010	0.3290	50
RPI DGP2 512	1	29,532	2500	0.010	1.6186	50
RPI MLP 32	16	117	500	0.005	0.0007	NA
RPI MLP 64	8	486	500	0.005	0.0007	NA
RPI MLP 128	4	1890	500	0.005	0.0006	NA
RPI MLP 256	2	7383	500	0.005	0.0006	NA
RPI MLP 512	1	29,532	500	0.005	0.0006	NA

**Table A2:** Overview of the data-driven models for the Theta dataset, their properties and time to train.

Dataset Model $2^{N}$	Row and Column Nr Divisible by	Nr of Datapoints	Nr Iterations	Learning Rate	Time Per Iteration (s)	Nr Inducing Points
Theta GP 32	16	36	500	0.020	0.0124	NA
Theta GP 64	8	156	500	0.020	0.0149	NA
Theta GP 128	4	675	500	0.020	0.0196	NA
Theta GP 256	2	2750	500	0.020	0.1964	NA
Theta GP 512	1	11,311	500	0.020	0.6781	NA
Theta DGP 32	16	36	5000	0.020	0.0515	36
Theta DGP 64	8	156	5000	0.020	0.0464	50
Theta DGP 128	4	675	5000	0.020	0.0546	50
Theta DGP 256	2	2750	5000	0.020	0.0857	50
Theta DGP 512	1	11,311	5000	0.020	0.4767	50
Theta DGP2 32	16	36	5000	0.010	0.0479	36
Theta DGP2 64	8	156	5000	0.005	0.0451	50
Theta DGP2 128	4	675	5000	0.005	0.0941	50
Theta DGP2 256	2	2750	5000	0.005	0.0855	50
Theta DGP2 512	1	11,311	5000	0.005	0.4960	50
Theta MLP 32	16	36	2000	0.005	0.0014	NA
Theta MLP 64	8	156	2000	0.005	0.0013	NA
Theta MLP 128	4	675	2000	0.005	0.0013	NA
Theta MLP 256	2	2750	2000	0.005	0.0013	NA
Theta MLP 512	1	11,311	2000	0.005	0.0014	NA

**Table A3:** Overview of the data-driven models for the Pillcam dataset, their properties and time to train.

Dataset Model $2^{N}$	Row and Column Nr Divisible by	Nr of Datapoints	Nr Iterations	Learning Rate	Time Per Iteration (s)	Nr Inducing Points
Pillcam GP 32	16	119	500	0.010	0.0111	NA
Pillcam GP 64	8	534	500	0.010	0.0138	NA
Pillcam GP 128	4	2070	250	0.010	0.2054	NA
Pillcam GP 256	2	8095	250	0.010	0.8548	NA
Pillcam DGP 32	16	119	5000	0.020	0.0523	50
Pillcam DGP 64	8	534	5000	0.020	0.0535	50
Pillcam DGP 128	4	2070	5000	0.020	0.0747	50
Pillcam DGP 256	2	8095	5000	0.020	0.2722	50
Pillcam DGP 512	1	31,686	1800	0.020	3.9022	50
Pillcam DGP2 32	16	119	5000	0.010	0.0561	119
Pillcam DGP2 64	8	534	5000	0.010	0.0849	150
Pillcam DGP2 128	4	2070	5000	0.010	0.4073	150
Pillcam DGP2 256	2	8095	5000	0.010	0.8257	150
Pillcam DGP2 512	1	31,686	2500	0.010	4.0370	150
Pillcam MLP 32	16	119	500	0.005	0.0006	NA
Pillcam MLP 64	8	534	500	0.005	0.0006	NA
Pillcam MLP 128	4	2070	500	0.005	0.0006	NA
Pillcam MLP 256	2	8095	500	0.005	0.0005	NA
Pillcam MLP 512	1	31,686	500	0.005	0.0007	NA
